# Effects of aqueous extracts of *Taraxacum Officinale* on expression of tumor necrosis factor-alpha and intracellular adhesion molecule 1 in LPS-stimulated RMMVECs

**DOI:** 10.1186/s12906-016-1520-3

**Published:** 2017-01-11

**Authors:** Ge Hu, Junjie Wang, Dong Hong, Tao Zhang, Huiqin Duan, Xiang Mu, Zuojun Yang

**Affiliations:** 1 Department of Animal Science and Technology, Beijing Agricultural College\Beijing Key Laboratory of Traditional Chinese Veterinary Medicine (TCVM), Beijing, 102206 People’s Republic of China; 2 Beijing Key Laboratory of Dairy Cow Nutrition, Beijing Agricultural College, Beijing, 102206 China

**Keywords:** Dandelion, TNF-α, ICAM-1, RMMVECs, Mastitis

## Abstract

**Background:**

Mastitis gives rise to big financial burden to farm industry (mainly dairy production) and public health. Its incidence is currently high and therefore, highly effective treatments for therapy, especially with natural products are required. *Taraxacum officinale* has been reported to use for anti-inflammation. However, its effect on endothelium during mastitis has not been reported.

**Methods:**

We firstly established inflammation experimental model of rat mammary microvascular endothelial cells (RMMVECs). We evaluated the effects of dandelion leaf aqueous extracts (DAE) on LPS-induced production of inflammatory mediators in RMMVECs by enzyme-linked immunosorbent assay and Western blot. We treated RMMVECs with 1 μg/ml LPS for 4 h and then incubated with 10, 100 and 200 μg/mL DAE for 4, 8, 12 and 24 h. The expression (mRNA and protein level) of targets (tumor necrosis factor-alpha (TNF- α) and Intracellular Adhesion Molecule 1 (ICAM1) was analyzed by employing real-time PCR and Western blots. The in vivo anti-inflammatory effect of DAE on mastitis within an *Staphylococcus aureus*-induced mouse model was also determined.

**Results:**

The obtained results showed that dandelion extracts at the concentration of 100 and 200 μg/mL could significantly inhibit both TNF-α and ICAM-1 expression in all time points checked while 10 μg/mL of dandelion only suppress both expression at 8 and 12 h post-treatment. The in vivo tests showed that the DAE inhibited the expression of TNF-α and ICAM-1 in a time-dependent manner.

**Conclusions:**

All results suggest that the endothelium may use as as a possible target of dandelion for anti-inflammation.

## Background

Mastitis, characterized as inflammation of the mammary gland and associated with decrease in milk secretion and quality [14], is a quite common problem for public health and dairy industry. For example, up to one third breastfeeding women have been reported for mastitis [8, 24] and bovine mastitis is a directly danger of the dairy industry [3]. Among many microbial and other factors inducing mastitis is the lipopolysaccharide (LPS) [16, 27], a component constituting the outer membrane of Gram-negative bacteria. LPS can activate the host tumor necrosis factor alpha (TNF-α) and trigger an inflammatory response [25]. Therefore, LPS is often used to induce mastitis in animal model [4, 5, 15]. TNF-α, is known as important target for antiinflammatory molecules, as an inflammatory mediators, is often activated and subsequently facilitates the transcription of a number of genes involved in inflammation [26, 30]. Additional, the expression of TNF-α, together other inflammatory factors such as interleukin 1 (IL-1), was also elevated even 3-4 h after post-challenge of LPS [3, 34]. Since activation of TNF-α and its corresponding downstream signaling pathways is mainly employed during initiation and development of mastitis [2, 12], new therapy with natural products targeting NTF-α activation is very promising.

*Taraxacum officinale* (common dandelion) has been used worldwide as herbal remedy to treat medical problems [6, 13, 28] including anti-inflammation for a long time. The anti-inflammatory effects have been confirmed in vitro or animal model by using extrats of *Taraxacum officinale* or its single components. *Taraxacum officinale* extracts (100 and 1000 μg/ml) was demonstrated to inhibit LPS-induced TNF-α production in rat astrocytes by inhibiting IL-1 production [9]. *Taraxacum officinale* extracts could induce cytotoxicity to hepatoma cell line HepG2 by increasing TNF-α production, leading to apoptosis [11]. Luteolin and luteolin-7-O-glucoside, two active components from *Taraxacum officinale* flower extracts, significantly suppressed the production of both inducible nitric oxide synthase (iNOS) and cyclooxygenase-2 (COX-2) in LPS activated-mouse macrophage RAW264.7 cells without introducing cytotoxicity [7]. Pre-treatment of two fractions from dandelion extracts with rich of TOP1 and TOP2, two polysaccharides, ameliorated the CCI4-induced hepatitis and related symptoms by partially inhibiting TNF-α and IL-1β expression in rat model [23]. However, the effect of *Taraxacum officinale* on LPS-induced activation of endothelium has not been investigated yet. Endothelium is a critical interface for bridging the signals between upstream and downstream during inflammation involved in mastitis. It provide a physical barrier between the blood stream and underlying vascular smooth muscle cells and play a critical role in the regulation of both vascular tone and changes in the growth and morphology of the vessel wall by maintaining a vital balance between the various dilating and constrictor processes. In this study, we investigated the effect of dandelion extract on the releasing of TNF-α and intracellular adhesion molecule 1 (ICAM-1) from cultured rat mammary microvascular endothelial cells in response to LPS-stimulation.

## Methods

### Reagents

Dulbecco’s modified Eagle’s medium, EC growth supplement, L-glutamine, penicillin/ streptomycin, fetal bovine serum (FBS) were obtained from Sigma (St. Louis, MO, USA). LPS was from Sigma (E. coli 055:B5, St. Louis, MO, USA) and diluted in sterile phosphate buffered saline (PBS) at a concentration of 1 μg/ml. RNA isolation kit was purchased from Invitrogen (Carlsbad, USA). Reverse-transcription kit was from Takara (Tokyo, Japan). anti-TNF-α monoclonal antibody, A biotinylated antibody, HRP-labeled avidin, anti-rat ICAM-1 antibody, horseradish peroxidase (HRP)-labeled rabbit anti-goat IgG, Anti β-actin monoclonal antibody and antibody recognizing rat CD34 were purchased from Boster (Wuhan, China).

### Preparation of dandelion leaf extracts

*Taraxacum officinale* were collected in Guizhou province, China, in October 2014 and identified by Prof. X.L. He in Northwest A&F University, China. A voucher specimen has been deposited in the Herbarium of College of Life Science of the university (the voucher specimen number 6446). They were cleaned and air dried for a week at 35–40 °C and then powdered in electric grinder. 100 g of the powdered *T. officinale* were extracted with sterile water for 4 h at 65 °C for three replicates and then concentrated to dryness by a vacuum freeze dryer (Chemical-free, Operon Co., Ltd., Korea). The dried extracts (DAE) were dissolved in maintenance medium (Dulbecco’s modified Eagle’s medium containing 2% FBS) prepare at a final concentration of 20 mg/ml.

### Cells grouping and processing

Sprague-Dawley rat (5 days postpartum) (Institute of Genetics and Developmental Biology, Chinese Academy of Sciences, Beijing, China), were killed by cervical dislocation and the mammary gland tissues was removed and washed with Hank’s buffer (Sigma; St. Louis, MO, USA) three times to remove residual blood and milk. The tissues was then peeled off using fine forceps, sheared into small pieces (about 1 mm3) with surgical scissors, and digested with 0.25% trypsin and 0.1% collagenase (Sigma; St. Louis, MO, USA) at 37 °C for 10 min. The digestion was terminated by adding FBS and the sample was centrifuged for 5 min. After discarding the supernatant, the remaining sediment was resuspended in Dulbecco’s modified Eagle’s medium containing 20% FBS, 1% EC growth supplement, 2% L-glutamine and 1% of both penicillin / streptomycin, and cultured on a 35-mm plate in an incubator at 37 °C under 5% CO_2_. The rat mammary microvascular endothelial cells (RMMVECs) migrated out of the tissue after 48–60 h, after which the tissue was removed.

**Fig. 1:**
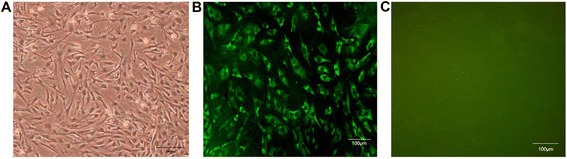
Validation of primary microvascular endothelial cells of rat mammary gland, at 95% confluency. light microscopy observation (400×) (**a**); cells immunostained with a fluorescein-conjugated anti-factor CD34 antibody (400×) (**b**) and with nonspecific rabbit immunoglobulin G as a negative control (**c**)

When the remaining cells had grown to confluency after 5–7 days, The RMMVECs were validated with purity of more than 95%, evaluated by immunostaining (Fig. 1).0.25 × 10^7^/well RMMVECs, with passage number between 2 and 3, were plated onto each well of 6-well plate and were kept under the conditions at 37 °C with 5% CO_2_ overnight. RMMVECs were pre-treated for 4 h with 1 μg/ml LPS, and then, dandelion solution with 10.0, 100.0 and 200.0 μg/ml were added to LPS-treated RMMVECs, respectively. Equal volume of water instead of dandelion solution was used as control. RMMVECs were harvested at different time point (4, 8, 12 or 24 h) after the addition of dandelion solution for further experiments. For statistical analysis, it was triplicate for each group. All procedures wereapproved by the Animal Care and Use Committee of Beijing University ofagriculture (Beijing, China).

### Real-Time PCR

RNAs were isolated from dandelion treated RMMVECs according the manufacture’s protocol (Invitrogen; Carlsbad, USA), with DNaseI treatment. 0.5 μg RNAs were used to generate cDNA by using the one-step RT-PCR kit (Takara; Tokyo, Japan). The quantitive real-time PCR reaction system (Applied Biosystems; Foster City, CA, USA) was prepared with 10 μL of qRT-PCR Mix, 0.4 μL of Forward/Reverse primer (5 μM), 1 μL cDNA and 8.2 μL sterilized double distilled water. β-Actin was used as internal standard gene in real-time PCR quantification method to relative quantify the expression of ICAM-1 and TNF-α. The qRT-PCR reaction was done with RT-PCR instrument (Applied Biosystems ABI PRISM® 7300) under the following conditions: Pre-denaturation was done at 95 °C for 10 min followed by 45 cycles including 30 s at 95 °C with 31 s at 61 °C; finally the following melting program was added according to the instrument default: 95 °C for 15 s, 60 °C for 30 s and 95 °C for 15 s, in order to verify that the specificity of the reaction and determine the expression performance of ICAM-1 and TNF-α cytokines. The primers used were synthesized by Shanghai Sangon Biological Engineering Technology & Services Co.,Ltd.; Shanghai, China and are shown in Table 1.

**Table 1:** Primer sequence of Real-time PCR related

Gene	Primer sequences	Fragment
TNF-α F	TGACCCCCATTACTCTGACC	152 bp
TNF-α R	TTCAGCGTCTCGTGTGTTTC	
ICAM-1 F	AACGACGCTTCTTTTGCTCTG	136 bp
ICAM-1 R	TCTTGCCAGGTCCAGTTCC	
β-actin F	GCTACAGCTTCACCACCACA	94 bp
β-actin R	GCCATCTCTTGCTCGAAGTC	

### Cytokine enzyme-linked immunosorbent assay (ELISA)

Cells in each groups were centrifuged and then collected the supernatants, and the supernatant was taken for experiment. The procedure for quantifying the concentration of TNF- α in cellular medium was carried out according to the instructions of the ELISA kit (R&D Systems; Minneapolis, USA). Briefly, 100-μL samples were added to the wells of a 96-well ELISA plate coated with anti-TNF-α monoclonal antibodies and mixed well. Blank control wells were set up and processed at the same time. The plate was sealed and then incubated at 37 °C for 90 min. The plate was washed four times with washing buffer. 100 μL diluted biotinylated antibody was added to each well and mixed thoroughly. The plate was sealed and incubated at 37 °C for 60 min. After washing for four times with washing buffer, a HRP-labeled avidin (100 μl) was added to each well and mixed. The plate was incubated at 37 °C for 30 min after being sealed. After washing, a colored liquid (100 μL) was added to each well and the plate was incubated at 37 °C in the dark for 15 min before 100 μL stop buffer was added to each well and mixed to stop the reaction.

The absorbance values of each well were measured at 450 nm as the optical densities (ODs). The OD values were used as ordinates, with the standard concentrations on the x-axis and the absorbance values on the y-axis, and a standard curve was drawn for these data. The sample concentrations were calculated according to the OD values of the samples based on the standard curve. Triplicate wells were set up for each group for statistical analysis. At least three independent experiments were repeated.

### Western blotting

RMMVECs were washed one time with pre-cold 1 × DPBS. 200 μL of RIPA lysis buffer was added into each well and incubated at 4 °C for 3-5 min. The cellular lysates were transferred into a 1.5 mL centrifuge tube and gently shaked once every 5–6 min on an oscillator for total 4 times. The cellular lysates were centrifuged at 10000 g for 3 min and the supernatant was taken to run SDS-PAGE; the separated proteins were transferred to PVDF membrane, and blocked with blocking solution (Cwbiotech; Beijing, China) on shaker table for 1 h at room temperature. After being washed with 1 × TBST (Cwbiotech; Beijing, China) three times, the PVDF membrane was incubated with goat anti-rat ICAM-1 antibody (R&D Systems; Minneapolis, USA) (1:2000) at 4 °C the overnight. The membrane was washed with TBST for three times and incubated with HRP-conjugated rabbit anti-goat IgG (1:5000) at 37 °C for 1 h. After washing, 1 ml DAB (Cwbiotech, Beijing, China) was used to develop color and the stripe gradation were analyzed, we use the PBS as blank control.

### In vivo anti- inflammatory test

Sixty adult female BALB/c mice (8–10 weeks old) were used in the present study and provided by the Center of Experimental Animals at Academy of Military Medical Sciences in China. The procedures were performed in accordance with the NIH Guide for the Care and Use of Laboratory Animals and approved by the Institutional Animal Care and Use Committee of Beijing Agricultural College. The female mice had just given birth to offspring and were lactating. Two abdominal mammary glands were stimulated with *Staphylococcus aureus*. The 50 ul of 10^9^ cfu/ml S*. aureus* was injected via the teat canal with a 100-μl microsyringe to induce mammary gland inflammation. DAE was dissolved in physiological saline for oral administration 4 g/kg × d for 9 days. The mice were fed food and water ad libitum in an air-conditioned room with a temperature maintained at 24 ± 1 °C. All of the mice were randomly divided into three groups as follows: 1) the *S. aureus* -induced mastitis group (SI), which included untreated mice with *S.aureus*-induced mastitis; 2) the DAE administration groups (DAEs), which were subjected to *S.aureus*-induced mastitis and oral administrated with DAE 4 g/kg × d for 9 days and 4) the blank control group (CG), which included untreated mice. The mice were euthanized with sodium pentobarbital. Mammary gland tissue was quickly harvested for further analyses.

### Histological analysis

Mammary gland tissues were fixed in 10% formalin for one week. Samples were obtained from embedded paraffin and deparaffinized with xylene and rehydrated with graded alcohol for staining analysis. The sections were stained with hematoxylin and eosin (H&E), and then visualized with a microscope (Olympus, Japan).

### Statistical analysis

Student’s *t*-test was performed using SPSS16.0 (SPSS Inc.). If *p* value is less than 0.05, difference between test group and the control group is considered to be statistically significant, while values of *p* < 0.01 were considered to be very significant.

## Results

### RMMVECs are identified by factor CD_34_

Since it has reported that bovine gland showed similar morphology with murine after being infected by *E. coli* [1], we used rat model of mastitis to mimic cow mastitis for following study, by using rat mammary microvascular endothelial cells. To validate the primary cells are indeed endothelial cells, we stained these cells with antibody recognizing CD34, a marker of endothelial cells, after being plated.

These RMMVECs show similar morphology under light microscopy after being plated (Fig. 1a). To further validate these cells are endothelial cells from rat, we stained these cells with fluorescein- conjugated rabbit anti-factor CD34 polyclonal antibody. As shown in Fig. 1b, over 95% of the isolated cells were positive for the CD34, while no staining with nonspecific rabbit immunoglobulin G (IgG) (Fig. 1c), indicating that these cultured cells in our preparation are endothelial cells, which are used for further experiments.

### TNF-α expression level in RMMVECs in response to LPS stimulation

**Fig. 2:**
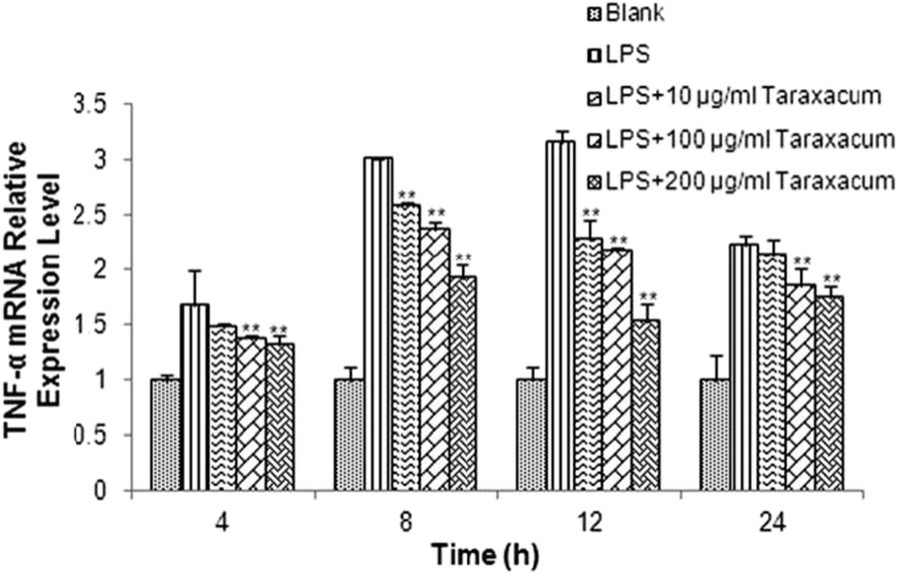
Expression of TNF-α mRNA in RMMVECs upon 1 μg/mL LPS stimulation in presence or absence of dandelion at different time point. 1 μg/mL LPS solution was used to stimulate RMMVECs and dandelion with 10, 100 and 200 μg/mL were added to RMMVECs at 4 h post LPS-stimulation. Quantitative real-time PCR was used to analyze the TNF-α mRNA expression at 4, 8, 12 and 24 h post-dandelion treatment. The relative expression of TNF-α were shown by normalizing the mRNA level of TNF-α to that of a housekeeping gene β-actin. Data are expressed as means ± SEM (n = 3 in each group). * *p* < 0.05 or ** *p* < 0.01 vs. LPS stimulating group at the same time point

It is well known that LPS enhanced TNF-α expression [2, 12]. To validate our RMMVECs, we first detecetd the TNF-α mRNA expression in RMMVECs in response to LPS stimulation. RMMVECs were harvested at different time point after stimulation and mRNA were isolated. qRT-PCR analysis showed that LPS did gradually induce TNF-α mRNA expression with peak induction between 12 and 24 h and went down but still significantly higher than basal expression level (*p* < 0.01) during the duration tests here as shown in Fig. 2, which indicates that LPS at the concentration of 1 μg/mL is able to increase the transcription of TNF-α in our RMMVECs. We then added dandelion aqueous extract with concentration at 10, 100 and 200 μg/mL to LPS-stimulated RMMVECs. The expression of TNF-α was significantly increased when compared to the control, in a dose dependent manner. Interestingly, dandelion at 10 μg/mL inhibit TNF-α expression at all time points tested with significantly reduction at 8 and 12 h post-treatment, respectively (Fig. 2). Therefore, dandelion is able to lower TNF-α mRNA expression in RMMEVs.

**Fig. 3:**
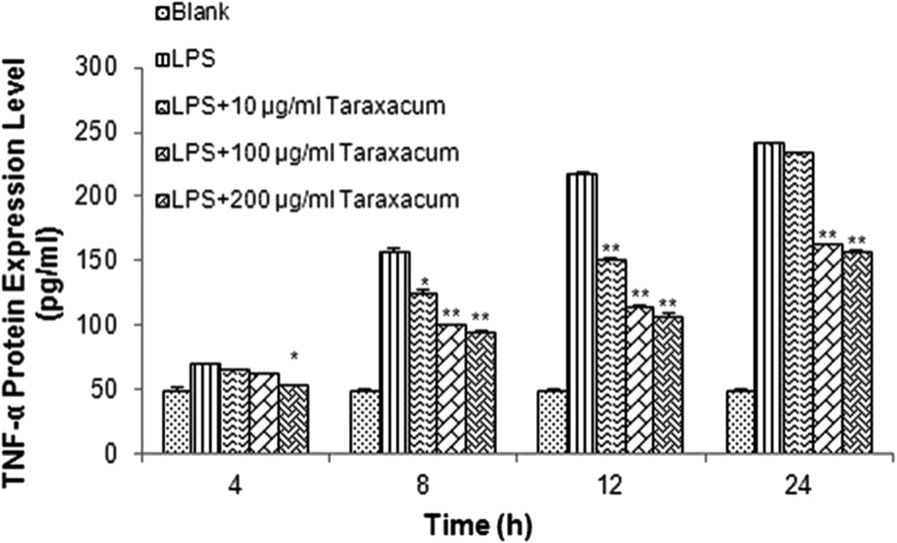
Level of TNF-α release by RMMVECs upon 1 μg/ml LPS stimulation in presence or absence of dandelion at different time point. 1 μg/mL LPS solution was used to stimulate RMMVECs and dandelion with 10, 100 and 200 μg/mL were added to RMMVECs at 4 h post LPS-stimulation. ELISA was used to analyze the release level of TNF-α protein into the medium at 4, 8, 12 and 24 h post-dandelion treatment. Data are expressed as means ± SEM (n = 3 in each group). * *p* < 0.05 or ** *p* < 0.01 vs. LPS stimulating group at the same time point

Since endothelial cells are able to release cytokines and chemokines upon inflammatory stimulation, we also tested the ability of RMMVECs to release TNF-α protein upon LPS stimulation for a continuously inflammatory response. ELISA was used to measure TNF-α concentration in medium to monitor the lease of TNF-α of RMMVECs to surrounding environments. As shown in Fig. 3, the TNF-α release did not show apparently different at early time (4 h) with control group, but the difference became dramatically higher at late time as the duration of LPS stimulation increased, which confirms that our RMMVEs is active for releasing cytokines and chemokines upon LPS stimulation. We also evaluated the effect of dandelion on the TNF-α release by RMMVECs. Similarly, TNF-α release were not significantly changed at 4 h post-treatment of dandelion (10 and 100 μg/mL) except 200 μg/mL dandelion was used (Fig. 3). However, dandelion treatment was able to significantly reduce the TNF-α release at late time points when higher concentration of dandelion aqueous extracts was used as seen in Fig. 3.

Based on these observation, we can conclude that relative high dose of dandelion treatment (i.e., 100 or 200 μg/mL) is able to inhibit the TNF-α transcription and protein release induced by LPS stimulation.

### ICAM-1 expression level in RMMVECs

Among major cell mediators is endothelial cell during inflammatory reaction. Besides the way to bridge the inflammatory response, endothelial cells express a set of adhesion molecules such as ICAM-1, whose expression are not detectable or low in physiological conditions, to mediate leukocytes recruitment [1]. TNF-α was reported to induce ICAM-1 expression [1]. Therefore, we examined the ICAM-1 expression, in both mRNA and protein level, in response to LPS stimulation in RMMVECs with/without dandelion treatment, to test whether ICAM-1 expression is affected by such stimulation and treatment.

**Fig. 4:**
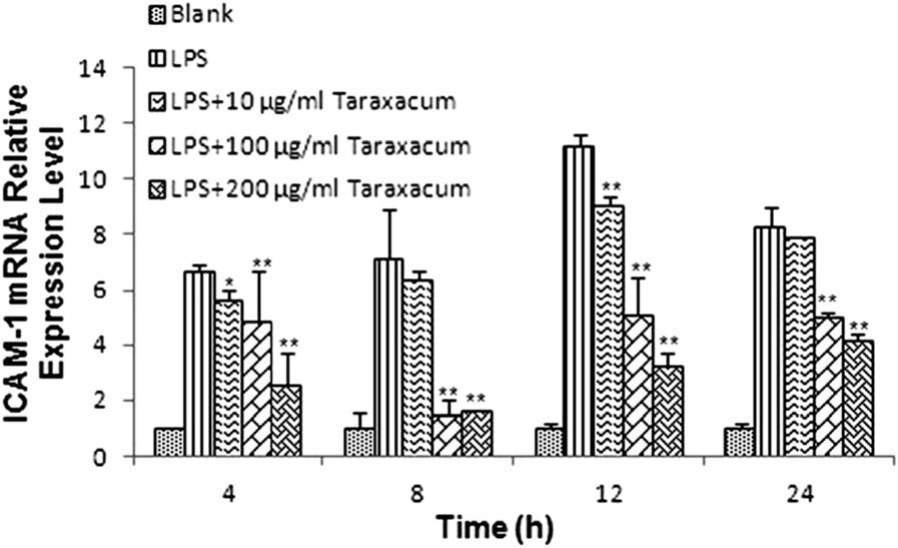
Expression of ICAM-1 mRNA in RMMVECs upon 1 μg/mL LPS stimulation in presence or absence of dandelion at different time point. 1 μg/mL LPS solution was used to stimulate RMMVECs and dandelion with 10, 100 and 200 μg/mL were added to RMMVECs at 4 h post LPS-stimulation. Quantitative real-time PCR was used to analyze the ICAM-1 mRNA expression at 4, 8, 12 and 24 h post-dandelion treatment. The relative expression of ICAM-1 was shown by normalizing the mRNA level of ICAM-1 to that of a housekeeping gene β-actin. Data are expressed as means ± SEM (n = 3 in each group). * *p* < 0.05 or ** *p* < 0.01 vs. LPS stimulating group at the same time point

**Fig. 5:**
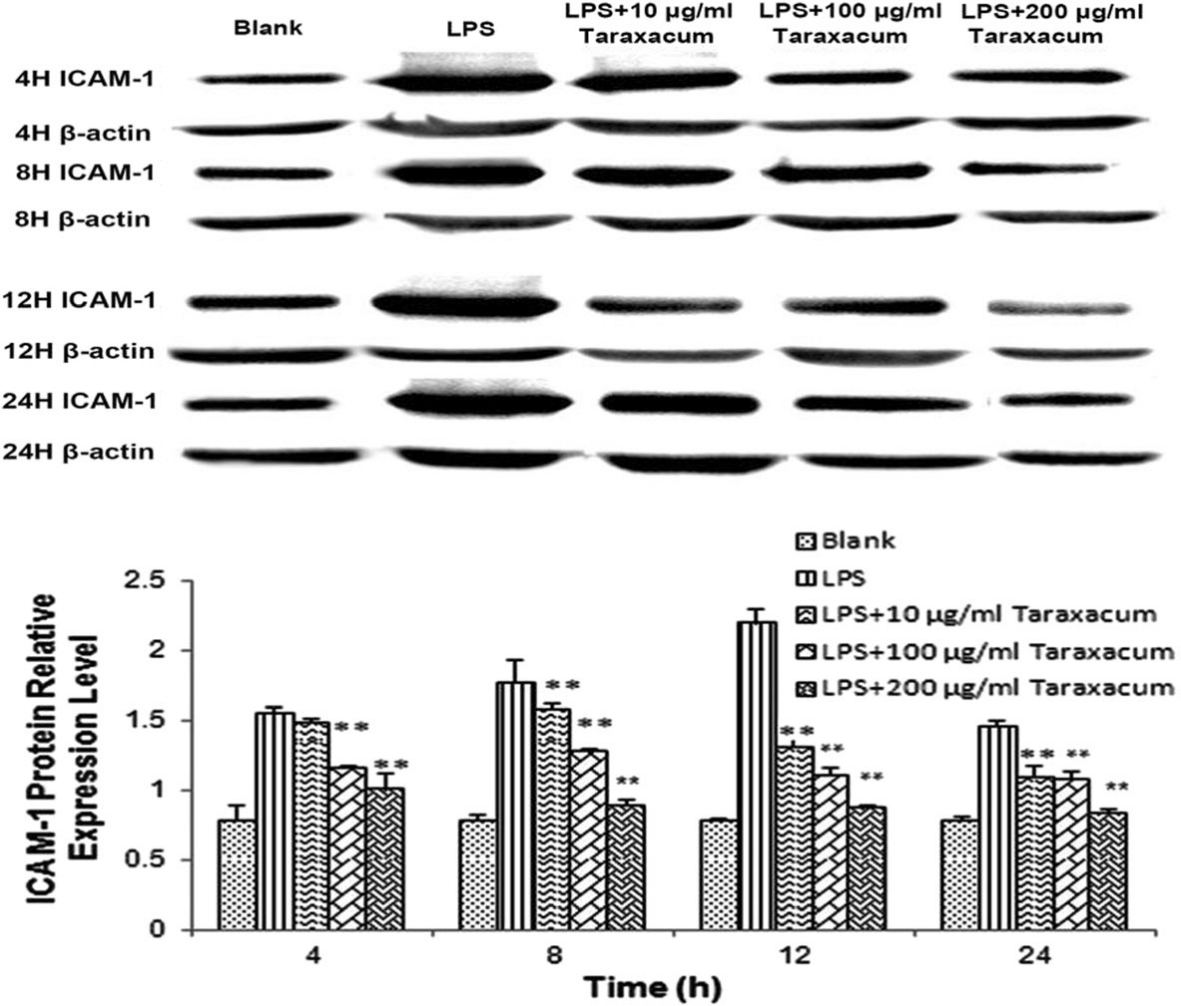
Expression of ICAM-1 mRNA in RMMVECs upon 1 μg/mL LPS stimulation in presence or absence of dandelion at different time point. 1 μg/mL LPS solution was used to stimulate RMMVECs and dandelion with 10, 100 and 200 μg/mL were added to RMMVECs at 4 h post LPS-stimulation. Quantitative real-time PCR was used to analyze the ICAM-1 mRNA expression at 4, 8, 12 and 24 h post-dandelion treatment. The relative expression of ICAM-1 was shown by normalizing the mRNA level of ICAM-1 to that of a housekeeping gene β-actin. Data are expressed as means ± SEM (n = 3 in each group). * *p* < 0.05 or ** *p* < 0.01 vs. LPS stimulating group at the same time point

qRT-PCR analysis showed that the ICAM-1 mRNA expression level within RMMVECs is steadily but significantly (*p* < 0.01) increasing within early time with highest at 12 h (about 12-fold) and then decreasing to 8-fold one day after stimulation, indicated in Fig. 4. Similar to the effect of dandelion on TNF-α mRNA expression in RMMVECs, low dose of dandelion (10 μg/mL) already showed its potential for reducing ICAM-1 mRNA expression in some experimental time points (Fig. 4), while dandelion at high doses (100 and 200 μg/mL) significantly inhibited ICAM-1 mRNA level at all time points, with highest inhibition at 8 h post-treatment. These data clearly show dandelion is able to repress LPS-induced ICAM-1 transcription in RMMVECs. Western blot was used to check whether the regulated transcription of ICAM-1 would lead to corresponding change in protein upon LPS stimulation and dandelion treatment. Interestingly, the expression level of ICAM-1 protein in RMMVECs of LPS group was also gradually increased with the action time, which reached the highest value at 12 h with statistical significance compared to that of the control group (*p* < 0.01), then decreased gradually to level of early time as seen in Fig. 5. Upon dandelion treatment, the expression level of ICAM-1 protein in each time period was decreased gradually even at lower dose; and the expression level was lower than that in LPS group (*p* < 0.01) (Fig. 5). The strongest inhibition was observed at 12 h post treatment compared to LPS group in regard of percentage and, of course, 200 μg/mL of dandelion aqueous extract gave the best result (Fig. 5).

### In vivo anti- inflammatory test

To determine the effect of DAE on *S.aureus* -induced mastitis, the expressions of TNF-α and ICAM-1were measured in mammary gland tissues. As showed in Table. 2 the TNF-α and ICAM-1 levels were elevated significantly in the IS group as compared to those in the CG group. In contrast, the TNF-α and ICAM-1 levels were significantly reduced (p <0.05) in the DAE-administered group.

**Table 2:** TNF-α and ICAM-1expression levels in the blood after treated with DAE (ng/mL)

Groups	GC	SI	DAE (DAY 1)	DAE (DAY 3)	DAE (DAY 6)	DAE (DAY 9)
TNF-α	4.46 ± 0.27	10.88 ± 1.25^**^	9.96 ± 0.89^**^	8.12 ± 0.65^*^	6.26 ± 1.26^*^	4.96 ± 0.92
ICAM-1	122.32 ± 10.54	156.56 ± 8.29^**^	150.38 ± 7.32^**^	142.53 ± 6.39^*^	130.15 ± 10.14	120.85 ± 10.78

*P < 0.05, **P < 0.01vs. compared with control

**Fig. 6:**
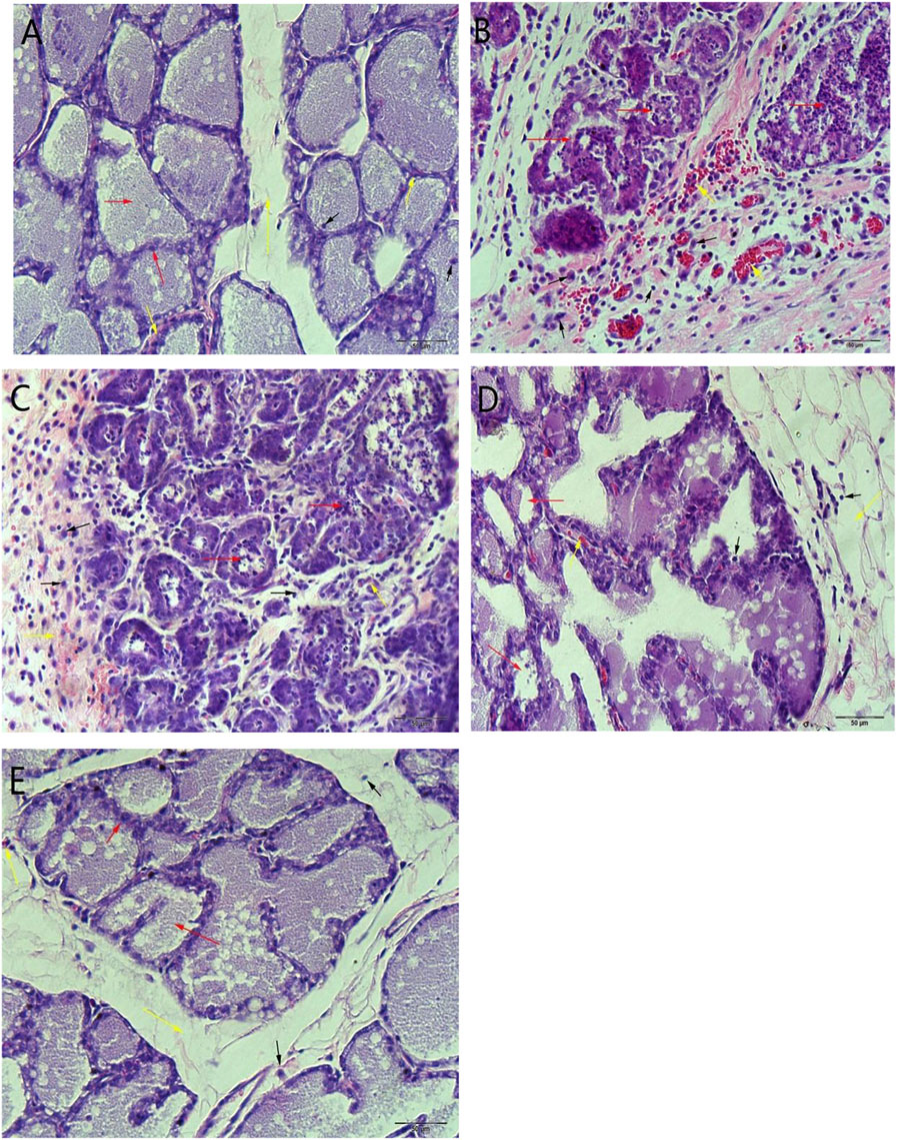
Histopathology of mammary tissue after *S.aureus*-induced mastitis (100×). **a** Mammary tissues of the CG group. **b** SI group (**c**), (**d**), (**e**) and DAEs treated at day 1, day 6 and day9 respectively

Mammary gland tissues were harvested on day1, 3, 6, and 9 after the oral administration. The tissue sections were subjected to H&E staining. No pathological lesions were observed in the CG group (Fig. 6a). In the SI group without drug treatment (Fig. 6b), the lobules of the mammary gland were incomplete, and inflammatory cells, including neutrophils and macrophages, were observed in the mammary acinus. The mammary epithelial cells were damaged, and the acini of the mammary glands were destroyed. Slight inflammatory injury was observed in the DAE group, as shown in Fig. 6c. These histopathological changes were ameliorated with sophocarpine administration. The effects of sophocarpine increased in a time-dependent manner (Fig. 6d, e). With time extended, inflammatory cell infiltration into mammary gland tissue declined, and lobule and acinus damage also decreased gradually, and the inflammatory injury proportion decreased gradually (Fig. 6). The change of the pathological proportion further showed the protective effect of sophocar DAE pine on the mammary gland tissues with *S.aureus*-induced mammitis.

## Discussion

It is well accepted that the LPS is able to elicit inflammatory response and is good inducer in mastitis of animal model [30]. TNF-α is one of numerous downstream targets of LPS in LPS-induced immunoreaction. TNF-α is key mediators during inflammatory responses via synergistically with interferon γ (IFN-γ) [20]. Therefore, inhibition of TNF-α expression would be one of primary targets for anti-inflammatory drugs. The present study demonstrated the anti-inflammatory effects of dandelion aqueous extract on LPS-induced rat mammary microvascular endothelial cells by reducing the both TNF-α secretion and ICAM-1 production/secretion, revealing a new possible target of dandelion executing its therapeutics for combating mastitis.

Dandelion has been long used for treat human diseases including inflammation [31]. Such anti-inflammation effect of dandelion may be via its effect on macrophage by reducing nitric oxide (NO), prostaglandin (PG) E2, TNF-α, IL-1β, IL-6, and cycloxygenase (COX)-2 [10, 22, 35]. Mammary epithelium also contributes innate immune response in mastitis induced by LPS [32, 33]. So far, the effect of dandelion extracts on endothelial cells is missing. Bovine endothelial cells released more antimicrobial peptides, including mlingual antimicrobial peptide (LAP) and bovine β-defensin 4 (BNBD4) in response to LPS stimulation and those peptides expression were dramatically elevated once with additional treatment with antibody recognizing TNF-α [2]. Therefore, endothelium may play important role during infection. Our study here showed anti-inflammatory effect of dandelion aqueous extract on endothelial cells, especially with dose of 100 μg/ml or higher concentration within 24 h treatment by reducing TNF-α and ICAM1 production/secretion. In inflammation, endothelium expresses more adhesion molecules such as ICAM-1 to regulate the adhesion of the circulating effector cells to endothelium. Once being activated or up-regulated, the TNF-α lease induced endothelial ICAM-1 during the elicitation of contact hypersensitivity [18, 19]. Therefore, our finding that dandelion can suppress production/secretion of TNF-α and ICAM1 reveals that the endothelium is also active target of dandelion for its anti-inflammation effect. This is the first report that dandelion extracts exert anti-inflammatory effect via endothelium.

Besides in vitro experiments, dandelion extracts or its active components have been demonstrated therapeutic effect in vivo. Pretreatment of macrophage cell line with 2.5-12.5 μg/ml taraxasterol, triterpene component of dandelion, inhibited TNF-α production 1 h prior LPS stimulation [35]; luteolin and chicoric acid, two components of dandelion, were found to suppress TNF-α and other cytokines secretion in macrophage cell line [22]; Methanol, ethyl acetate, and chloroform fractions of dandelion leaves showed positive anti-inflammatory effect on LPS stimulated microphage cell line. Furthermore, aqueous extract of dandelion leaf have been reported to ameliorate oxidative stress by reducing TNF-α expression in carbon-tetrachloride induced liver injury [21] or in LPS-induced acute lung injury [17]. TOP1 and TOP2, two polysaccharides from dandelion, were showed similar results in same liver injury model [23]. Taraxacum officinale extracts (10 mg/kg) also protected rats from cholecystokinin-octapeptide-induced acute pancreatitis [29]. Treatment of rat model of acute pancreatitis, challenged with 75 μg/kg cholecystokinin, with 10 mg/kg Taraxacum officinale significantly reduced the expression of both IL-6 and TNF-α along with reduction of the ratio of the pancreatic weight to body weight and increase of both heat shock protein (HSP) 60 and HSP70 level in pancreatis [29].

## Conclusion

The results reported here provide a possibility to extend our observations in animal model of mastitis. It would be interesting to find out which components of dandelion aqueous extract are active in elicit anti-inflammation effect on endothelium in LPS-induced mastitis model and which is most among them.

## Additional files

**Additional file 1:** Validation of primary microvascular endothelial cells of rat mammary gland, at 95% confluency.

**Additional file 2:** Expression of TNF-α mRNA in RMMVECs upon 1 μg/mL LPS stimulation in presence or absence of dandelion at different time point.

**Additional file 3:** Level of TNF-α release by RMMVECs upon 1 μg/ml LPS stimulation in presence or absence of dandelion at different time point.

**Additional file 4:** Expression of ICAM-1 mRNA in RMMVECs upon 1 μg/mL LPS stimulation in presence or absence of dandelion at different time point.

**Additional file 5:** Expression level of ICAM-1 by RMMVECs upon 1 μg/mL LPS stimulation in presence or absence of dandelion at different time point.

## Abbreviations

CD34: Clusters of differentiation
DAE: Dandelion leaf aqueous extracts
FBS: Fetal bovine serum
ICAM-1: Intracellular adhesion molecule 1
LPS: Lipopolysaccharide
RMMVECs: Rat mammary microvascular endothelial cells
RT-PCR: Quantitative real–time polymerase chain reaction
TNF- α: Tumor necrosis factor-alpha
